# Azacitidine in Combination with Venetoclax Maintenance Post-allogeneic Hematopoietic Stem Cell Transplantation in T Cell Acute Lymphoblastic Leukemia

**DOI:** 10.1007/s44228-022-00019-1

**Published:** 2023-01-03

**Authors:** Mona Ali Hassan, Nour Moukalled, Jean El Cheikh, Ali Bazarbachi, Iman Abou Dalle

**Affiliations:** Bone Marrow Transplantation Program, Department of Internal Medicine, American University of Beirut, Beirut, Lebanon

Relapsed/refractory (R/R) T-cell acute lymphoblastic leukemia (T-ALL) is an aggressive disease with few available salvage options, which carries a dismal outcome, with only 10% of patients surviving at 5 years [1]. Allogeneic hematopoietic stem cell transplantation (allo-HSCT) efficacy in R/R ALL remains questionable as up to 40–45% of recipients of HLA-identical sibling, and approximately 35% of recipients of unrelated donor transplants will relapse post allo-HSCT [2]. The risk of relapse is usually greatest in the first year after allo-HSCT, with half of those relapses occurring within 6 months of transplant [3]. One of the potential therapeutic strategies to decrease the risk of relapse post allo-HSCT in such high-risk disease is post-transplant maintenance treatment. Post-transplant maintenance in acute myeloid leukemia (AML) has long been debated with recent implementation by transplant centers, mostly with the use of hypomethylating agents (HMAs) [4] and FLT3 inhibitors [5]. In T-ALL, this concept has not been explored, yet, with limited available data [6]. In the French Group of Research on Adult ALL (GRAALL) 2003–2005 trial, analysis of 143 adult patients with T-ALL has identified different profiles, including hypermethylation of tumor suppressor genes suggesting a potential rationale for the use of HMAs such as 5-azacitidine. Multiple case series described the effectiveness of venetoclax in combination with chemotherapy or HMAs in the treatment of R/R T-ALL [7–9]. This has paved the way for the rationale of using HMAs and venetoclax as post-transplant maintenance in high-risk T-ALL.

Here, we present four adult patients diagnosed with high-risk T-ALL with a median age of 34 years (range, 30-45 years), a patient with early T-precursor (ETP) ALL, another one in second complete remission (CR2), a third with detectable measurable residual disease (MRD) post induction, and a patient with near ETP-ALL (Table 1), all of whom received post allo-HSCT maintenance with, low-dose azacitidine at 32 mg/m^2^ and venetoclax 400 mg daily (or 100 mg daily in combination with voriconazole) for 5 days, every 28 days. The median duration of maintenance treatment was 12.75 months (range, 9.5-23.5 months), with a very good safety profile. After a median follow up of 15 months from the date of allo-HSCT (range, 12-25 months), all patients remain in CR.

The first patient was diagnosed with mature type T-ALL with deletion (7) at the age of 30. He received Hyperfractionated Cyclophosphamide, Vincristine, Adriamycin, and Dexamethasone (hyper-CVAD) for 8 cycles, from May till December 2017, followed by maintenance with 6-mercaptopurine, vincrisitine, methotrexate, and prednisone (POMP) for 2 years. He was in continuous CR until December 2019 when bone marrow evaluation showed relapsed disease. He then received induction phase of augmented Berlin-Frankfurt-Münster regimen (BFM) after which he achieved CR with negative MRD by T-cell receptor (TCR) rearrangements. The patient then received one cycle consolidation which was completed in March 2020 after which he underwent full matched related allo-HSCT with clofarabine-total body irradiation (TBI) 4 Gy as conditioning and in-vivo T-cell depletion by Thymoglobulin (ATG). He was MRD negative at transplant. At day 42 post transplant, patient was started on maintenance treatment with 5-azacitidine and venetoclax. At day 100 he was in CR, with 99.5% donor chimerism, and undetectable MRD by TCR rearrangement. His transplant was only complicated with skin graft-versus-host disease (GVHD) managed by topical steroids. Patient is currently 25 months post transplant, remains in CR, continuing on maintenance, which has been well tolerated with no major hemtologic toxicities.

The second case was diagnosed at 31 years with ETP-ALL, with mutant NOTCH1 and EZH2, and normal karyotype. He received induction with hyper-CVAD for 4 cycles but still had detectable MRD by TCR rearrangements, so he had reinduction with augmented BFM therapy plus venetoclax. He continued with asparaginase-based treatment as consolidation, achieved complete molecular response by TCR rearrangement, and, he then had a haploidentical HSCT with fludarabine-TBI conditioning, and on day 53 post transplant, patient was started on 5-azacitidine plus venetoclax maintenance. At day 100 post transplant, TCR rearrangements MRD was detectable, thus he received donor lymphocyte infusion (DLI) for two doses, which was complicated by gastrointestinal and skin GVHD, responsive to systemic steroids. Currently this patient is 17 months post transplant with chronic skin GVHD continuing on maintenance treatment, with no hematologic toxicities, with last disease evaluation showing continuous CR with undetectable MRD (Table 1).

**Table 1:** Summary for the four patients who received 5-azacitidine and venetoclax maintenance post-transplant

Disease subtype	Status at transplant	Transplant type	Conditioning regimen	Days to azacitidine treatment (days)	Status at last F-up	CR duration post allo-SCT
T-ALL—30 yo male	CR2 molecular MRD negative	Full Match related donor	Clofarabine-TBI 4 Gy	42	Alive and in CR MRD negative	+ 25 months
ETP-ALL—31 y.o male	CR1,molecular MRD negative for TCR	Haplo-SCT	Fludarabine-TBI-8GY	53	Alive in CR MRD negative	+ 17 month
T-ALL—45 yo male	CR1 molecular MRD negative for TCR	Full Match related donor	Clofarabine-TBI 8 Gy	115	Alive and in CR MRD positive	+ 13 months
Near ETP-ALL—37 yo male	CR1 molecular MRD negative	Full Match related donor	Clofarabine- TBI 8 Gy	46	Alive and in CR MRD negative	+ 12 months

*T-ALL* T acute lymphoblastic leukemia, *ETP* early T precursor, *CR* complete remission, *MRD* measurable residual disease, *TCR* T-cell receptor, *SCT* stem cell transplantation, *TBI* total body irradiation

The 3rd case was diagnosed with T-ALL at 45 years of age with complex cytogenetics, EZH2, KIT and TET2 mutations. He received hyper-CVAD induction after which he achieved CR with detectable MRD. He then received Capizzi regimen with  venetoclax after which he had undetectable MRD, and underwent a matched-related allo-HSCT with clofarabine plus TBI conditioning. His post-transplant course was complicated by mild skin GVHD. His maintenance treatment started at day 115 post allo-HSCT, he has been on this therapy for 13 months to date, with very good tolerance. His last bone marrow evaluation showed continuous CR with full donor chimerism but detectable TCR MRD for which the patient received two DLI doses complicated by liver GVHD responsive to systemic corticosteroids.

The 4th case was 37 at diagnosis with near ETP ALL with normal karyotype, ASXL1, NOTCH1, and TET2 mutations. He received an induction according to the Group of Research on Adult ALL (GRAALL) protocol. He achieved CR, with undetectable MRD, then completed 2 consolidation cycles and early intensification followed by a matched-related allo-HSCT with clofarabine and TBI 8 Gy conditioning. He started post-transplant 5-azacitidine maintenance  daily for 5 days with venetoclax at 400 mg daily for 5 days at day 46 post transplant, with no hematologic toxicity. He is currently 12 months post-transplant and is still in CR with undetectable MRD.

Despite the advances in the treatment of B-cell ALL including novel agents such as blinatumomab, inotuzumab ozogamicin, among others, little progress has been seen in T-ALL. Nelarabine is the only approved drug in R/R T-ALL with an overall response rate of 50%, but with associated hematologic and neurological toxicities [10]. Allo-HSCT continues to be the main potentially curative strategy in this setting, given the graft-versus-leukemia (GVL) effect.

T lineage ALL cells express different levels of HLA-class II antigens, which may serve as targets for GVHD and GVL effect [11]. Accordingly, prophylactic DLI to boost the GVL effect was studied and its efficacy was demonstrated in a multicenter study of 123 advanced-stage acute leukemia patients which showed that the 2-year cumulative incidence of relapse was significantly lower in the preemptive or prophylactic DLI group (46%) than in the non-DLI group (66%) [12] but its efficacy remains limited due to the poor response to the GVL effect compared to the rapidly progressing leukemic burden and the risk of developing severe GVHD [13].

Both HMAs and the BCL-2 inhibitor venetoclax possess significant antitumor activity effects against acute myeloid leukemia/myelodysplastic syndrome and their efficacy in R/R T-ALL has been demonstrated in multiple case series [14]. T-ALL exhibited high in-vitro and in-vivo sensitivity to the BCL-2 inhibitor, venetoclax in correspondence with high levels of BCL-2 [15]. In the clinical setting, robust data is inexistent regarding post-transplant maintenance in patients diagnosed with T-ALL. A recent Chinese study evaluated the use of low dose decitabine as maintenance post-transplant in ALL, with promising results in those with T-ALL, as none of seven treated patients have relapsed [16].

These encouraging results along with our case series of successful maintenance treatment with 5-azacitidine and venetoclax in T-ALL are worth exploring in larger prospective trials. This case series presents a strong foundation for further prospective studies that could assess the efficacy of combining HMAs with BCL-2 inhibitors, not only in the post-transplant setting but also for T-ALL patients who fail to respond to standard treatment options.

## Declarations

### Availability of Data and Material

Data are available upon request.
